# Diagnosis-to-Treatment Interval Reflects Clinical Urgency and Disease Burden in T-Lymphoblastic Lymphoma: A Multicenter Cohort Study

**DOI:** 10.3390/jcm15166408

**Published:** 2026-08-19

**Authors:** Jin Chai, Yunyan Sun, Xuewen Lei, Linjun Zhao, Jie Chen, Wenhui Zhang, Yue Wang, Ni An, Lingyan Ping, Yuqin Song, Hui Yu

**Affiliations:** 1 Key Laboratory of Carcinogenesis and Translational Research (Ministry of Education), Department of Lymphoma, Peking University Cancer Hospital & Institute, Beijing 100142, China; chaijin19980702@163.com (J.C.); xjtucj@163.com (J.C.); kcacoz@163.com (W.Z.); wy1137502485@163.com (Y.W.); anni202121@163.com (N.A.); dingdingply1981@pku.edu.cn (L.P.); 2 Peking University Cancer Hospital Yunnan Hospital—Yunnan Cancer Hospital, The Third Affiliated Hospital of Kunming Medical University, Kunming 650118, China; syy0124@sina.com; 3 Inner Mongolia Autonomous Region Cancer Hospital, The Affiliated People’s Hospital of Inner Mongolia Medical University, Hohhot 010020, China; xuewen.lei6@outlook.com; 4 Lymphoma Unit, Peking University International Hospital, Beijing 102200, China; linjun_88@126.com

**Keywords:** T-lymphoblastic lymphoma, diagnosis-to-treatment interval, clinical urgency, disease aggressiveness, treatment response

## Abstract

**Objective:** Diagnosis-to-treatment interval (DTI) has been investigated in several lymphoma subtypes, but its significance in T-lymphoblastic lymphoma (T-LBL) remains unclear. We evaluated whether DTI reflects clinical urgency, disease burden and early outcomes in newly diagnosed T-LBL. **Methods:** We retrospectively analyzed 220 patients with newly diagnosed, mass-dominant, and histologically diagnosed T-LBL treated at four centers between 2003 and 2025. DTI was operationally defined as the interval from diagnostic biopsy to first systemic anti-lymphoma therapy and was primarily analyzed as a continuous variable per 7-day increase. Descriptive DTI groups were <15, 15–29 and ≥30 days. **Results:** Median DTI was 20 days (interquartile range [IQR], 14–32). Shorter DTI was associated with advanced stage, elevated lactate dehydrogenase (LDH), multiple extranodal involvement, central nervous system (CNS) involvement, B symptoms, lower hemoglobin and higher International Prognostic Index (IPI) score. CNS involvement remained independently associated with DTI < 15 days. In adjusted analyses, each 7-day increase in DTI was associated with lower odds of early treatment failure within 2 years (odds ratio [OR] 0.72, 95% confidence interval [CI] 0.58–0.88), lower hazards of progression-free survival (PFS) events (hazard ratio [HR] 0.84, 95% CI 0.74–0.94) and lower mortality (HR 0.84, 95% CI 0.73–0.98), but not complete response. **Conclusions:** Shorter DTI appears to reflect clinical urgency and disease burden rather than a causal effect of early treatment.

## 1. Introduction

T-lymphoblastic lymphoma (T-LBL) is an aggressive precursor T-cell neoplasm that frequently presents with advanced-stage disease, mediastinal involvement, extranodal dissemination and rapid clinical progression [1,2,3,4,5]. Although intensive acute lymphoblastic leukemia-like regimens have improved outcomes, a substantial proportion of patients still experience early treatment failure or disease progression [2,5,6,7,8,9]. Conventional prognostic assessment in T-LBL is largely based on baseline clinical features, such as stage, lactate dehydrogenase (LDH), performance status, extranodal involvement, central nervous system (CNS) involvement and composite risk scores [2,10]. However, these variables may not fully capture the clinical urgency encountered in real-world practice at the time treatment decisions are made [11].

Diagnosis-to-treatment interval (DTI), defined as the time from diagnosis to initiation of systemic therapy, has been investigated in several lymphoma subtypes [12,13,14,15,16,17,18]. In indolent or moderately aggressive diseases, prolonged DTI may raise concern about delayed care [14,18,19,20]. In contrast, studies in aggressive lymphomas have suggested a paradoxical association whereby shorter DTI is linked to inferior outcomes [10,12,13,15,17]. This observation is generally interpreted as reflecting confounding by indication: patients with more symptomatic, advanced or biologically aggressive disease are treated more rapidly, and their poorer outcomes are driven by the underlying disease rather than by early treatment itself [21]. Therefore, DTI may function less as a simple measure of treatment delay and more as a real-world surrogate of clinical urgency and disease aggressiveness [19,21].

This concept may be particularly relevant to T-LBL, in which treatment decisions are often made under substantial clinical urgency. Unlike many lymphoma subtypes, T-LBL frequently presents with bulky mediastinal disease, extensive extranodal dissemination, marrow or CNS involvement, high tumor burden, or clinical instability, all of which may prompt immediate treatment initiation [1,2]. In such settings, DTI may capture an aspect of real-world clinical decision making that is not fully represented by conventional baseline prognostic variables. However, whether short DTI identifies a clinically urgent and biologically aggressive subgroup of T-LBL has not been systematically evaluated.

In this multicenter retrospective cohort study, we investigated DTI in 220 patients with newly diagnosed T-LBL. We aimed to describe the distribution of DTI, identify baseline clinical features associated with short DTI, and evaluate its association with treatment response, early treatment failure, progression-free survival (PFS) and overall survival (OS). We hypothesized that shorter DTI would be associated with higher baseline disease burden and inferior outcomes, supporting its role as a real-world marker of clinical urgency and disease aggressiveness rather than a causal determinant of prognosis.

## 2. Materials and Methods

### 2.1. Study Population

We retrospectively reviewed patients with newly diagnosed T-LBL treated at four centers between 2003 and 2025. Patients were eligible if information was available on baseline clinical characteristics, date of diagnostic biopsy or tissue sampling, date of first systemic anti-lymphoma therapy, first-line treatment, treatment response, progression status, survival status and follow-up. Patients were excluded if key date information or survival outcomes were unavailable, or if systemic anti-lymphoma therapy had already been initiated before referral to the participating centers. A total of 220 patients were included.

This study was conducted in accordance with the Declaration of Helsinki and was approved by the Institutional Review Board of Peking University Cancer Hospital (approval number: 2025KT85). The requirement for informed consent was waived because of the retrospective nature of this study and the use of de-identified data.

### 2.2. Definition of Diagnosis-to-Treatment Interval

DTI was operationally defined as the interval from diagnostic biopsy or tissue sampling to initiation of first systemic anti-lymphoma therapy. Biopsy date was used as the primary starting point because it was consistently documented across centers and eras. The date of final pathological diagnosis was collected when available. In available records, the median biopsy-to-final-pathology interval was 7 days (IQR, 6–10; range, 2–40). Pathology-date-based DTI was available for 162 patients and was evaluated in a sensitivity analysis. Pre-phase corticosteroid status was available for 59 patients: 27 received corticosteroids and 32 did not.

DTI was primarily analyzed as a continuous variable, with effect estimates reported per 7-day increase. For descriptive comparisons and visualization, patients were also grouped as <15 days, 15–29 days and ≥30 days. These categories were informed by the observed DTI distribution, restricted cubic spline patterns and clinical interpretability, and were not intended to represent predefined biological cut-offs.

### 2.3. Clinical Variables, Treatment Response and Endpoints

Baseline variables included age, sex, Ann Arbor stage, LDH, Ki-67 index, B symptoms, Eastern Cooperative Oncology Group (ECOG) performance status, number of extranodal sites, bone marrow involvement, bone marrow blast percentage when evaluable, CNS involvement, mediastinal involvement, bulky disease and International Prognostic Index (IPI). Baseline WBC, hemoglobin, platelet count, albumin and uric acid were available in 176 patients. Treatment variables included first-line regimen and consolidative autologous or allogeneic hematopoietic stem cell transplantation.

Treatment responses recorded at participating centers were retrospectively harmonized as complete response (CR), partial response (PR), stable disease (SD) or progressive disease (PD) using Lugano 2014 definitions applied to the clinical and imaging records available for each treatment era [22]. Overall response was defined as CR plus PR. Early treatment failure was defined as progression, relapse or death within 2 years after first systemic therapy. Patients with progression, relapse or death within 2 years were classified as failures, whereas event-free patients observed for at least 2 years were classified as non-failures. Event-free patients with shorter follow-up were not assigned a binary 2-year outcome; they remained in PFS and OS analyses and were censored at their last follow-up.

The primary survival endpoint was PFS, defined as the time from initiation of first systemic therapy to progression, relapse, death from any cause or last follow-up. OS was defined as the time from initiation of first systemic therapy to death from any cause or last follow-up. Treatment response, overall response rate and early treatment failure were evaluated as secondary or exploratory endpoints. In sensitivity analyses, PFS and OS were recalculated from the diagnostic biopsy date.

### 2.4. Statistical Analysis

Continuous variables were summarized as medians with interquartile ranges and compared using the Mann–Whitney U test or Kruskal–Wallis test, as appropriate. Categorical variables were summarized as frequencies and percentages and compared using the χ^2^ test or Fisher’s exact test. Tests for trend across ordered DTI groups were performed for selected variables with clinically meaningful ordinal patterns.

Clinical factors associated with short DTI were evaluated using logistic regression, with DTI < 15 days as the outcome. Associations of DTI with CR and early treatment failure were assessed using logistic regression models. PFS and OS were estimated using the Kaplan–Meier method and compared using the log-rank test. Cox proportional hazards models were used to evaluate associations between DTI and survival outcomes. The primary models treated DTI as a continuous variable per 7-day increase; categorical DTI models were presented for descriptive and clinically interpretable comparisons. Odds ratios, hazard ratios, 95% confidence intervals and *p* values were reported.

For the exploratory logistic model of DTI < 15 days, variables were selected using clinical relevance plus univariable *p* < 0.10 and comprised stage, LDH, B symptoms, extranodal involvement > 1 site and CNS involvement. Outcome models for CR, early treatment failure, PFS and OS used a consistent a priori panel: age, stage, ECOG, LDH, extranodal involvement > 1 site, bone marrow involvement and CNS involvement. Core model variables were complete in all 220 patients; supplementary laboratory, pathology-date and pre-phase variables were analyzed using available cases without imputation. Post-treatment transplantation was not entered as a fixed baseline covariate because of time-dependent selection and immortal time bias.

Because most patients were treated at the lead center and two centers contributed few patients, the center was not included in primary multivariable models to avoid instability. Sensitivity analyses excluded DTI > 60 or >90 days, restricted the cohort to BFM-90-treated patients, calculated survival from biopsy date, adjusted for diagnosis year, recalculated DTI from final pathology date, excluded patients with documented pre-phase corticosteroid use, and restricted the cohort to 153 patients with no marrow involvement or a documented marrow blast percentage < 25% (excluding 54 with ≥25% and 13 with an unknown percentage). All statistical tests were two-sided, and *p* < 0.05 was considered statistically significant. Analyses were performed using R software (version 4.2.0).

## 3. Results

### 3.1. Patient Characteristics and DTI Distribution

**Figure 1:**
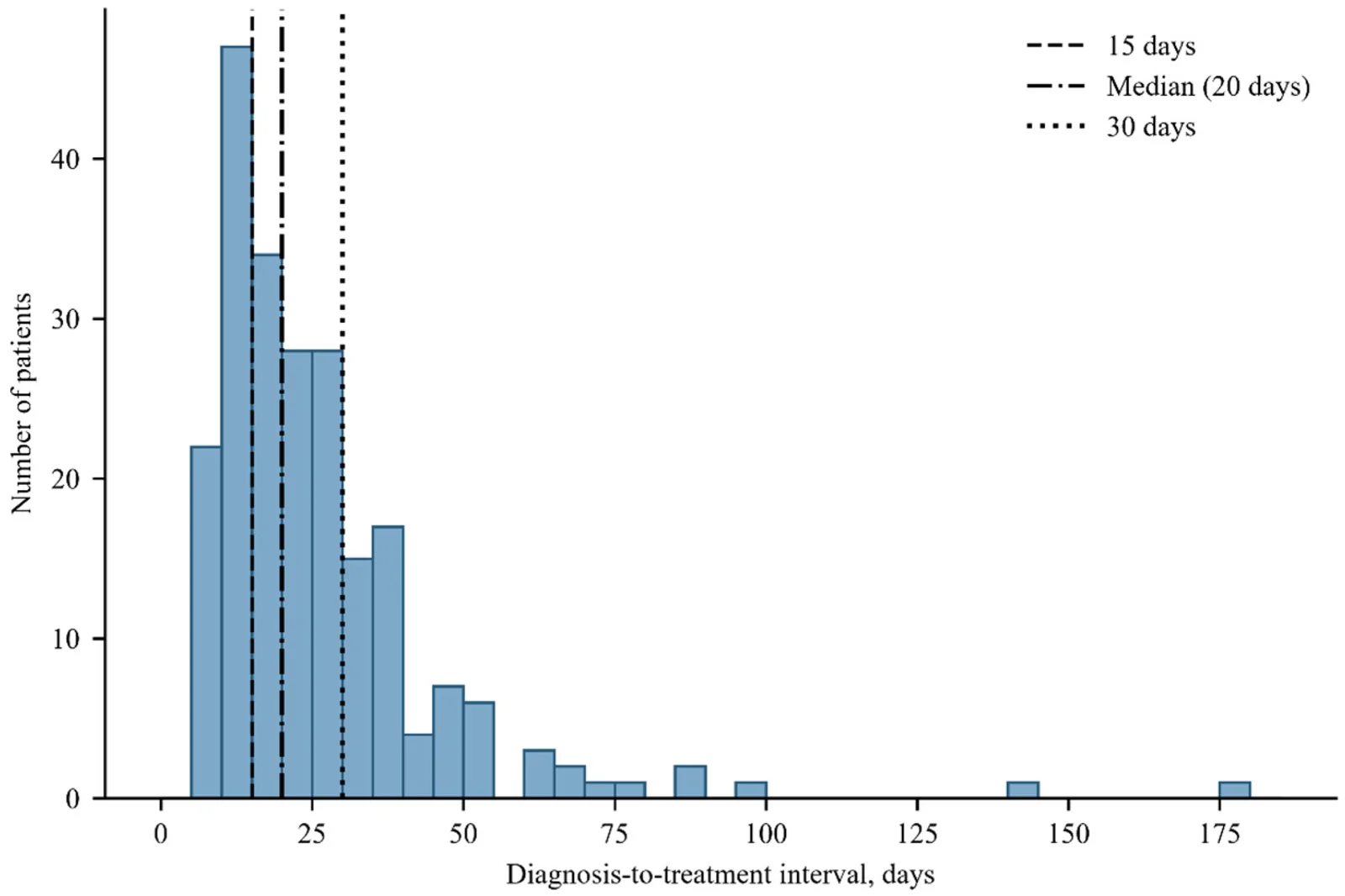
**Distribution of diagnosis-to-treatment interval.** The histogram shows DTI in days with reference lines at 15 days, the cohort median of 20 days, and 30 days. Line types distinguish the three reference points for black-and-white printing.

Of 236 patients screened, 220 were included: Peking University Cancer Hospital, 154 (70.0%); Yunnan Cancer Hospital, 56 (25.5%); Inner Mongolia Cancer Hospital, 3 (1.4%); and Peking University International Hospital, 7 (3.2%) (Supplementary Figure S1). The median age was 27 years (range, 8–69). The median DTI was 20 days (IQR, 14–32 days). Sixty-nine patients (31.4%) started treatment within 15 days, 90 (40.9%) between 15 and 29 days, and 61 (27.7%) at 30 days or later (Figure 1). Bone marrow involvement was present in 110 patients (50.0%). Among 97 with evaluable marrow flow cytometry or aspiration data, 54 had blasts ≥ 25%. Baseline characteristics according to the DTI group are summarized in Table 1.

### 3.2. Baseline Disease Burden According to DTI

**Figure 2:**
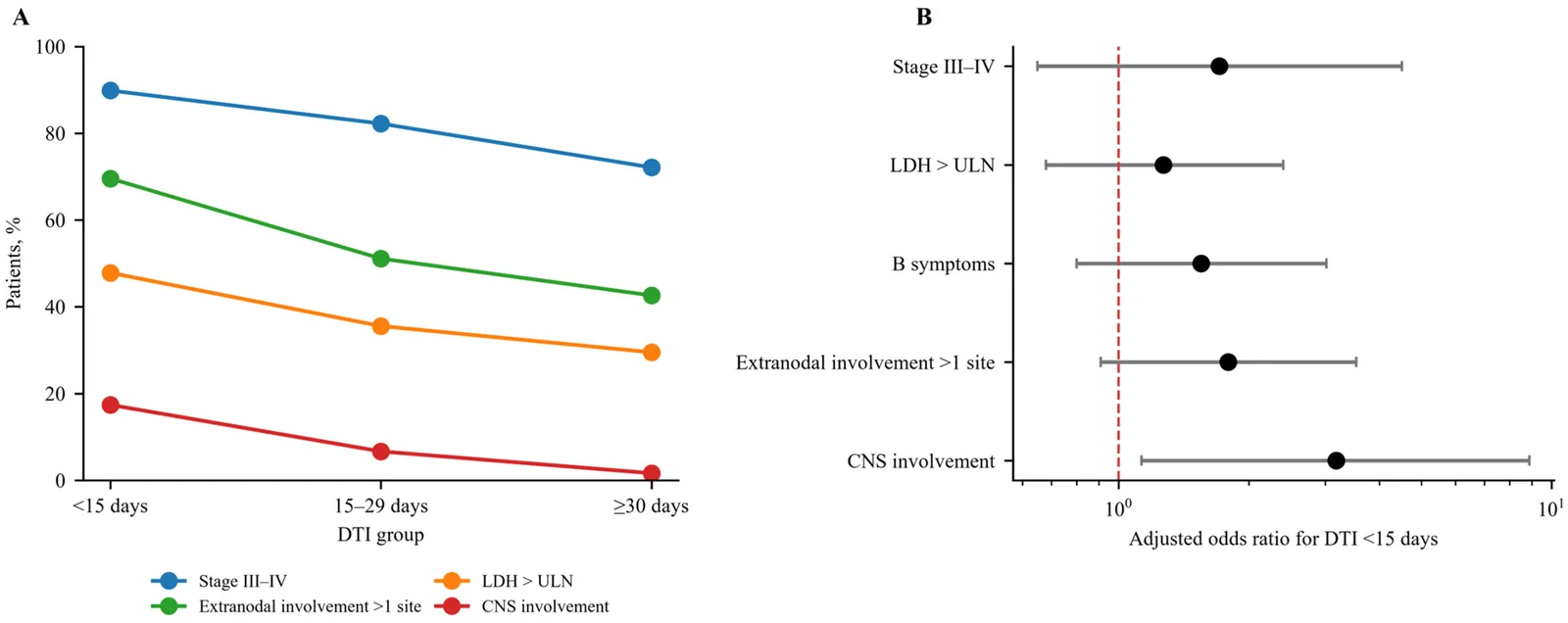
**Baseline disease burden and clinical factors associated with DTI < 15 days.** Panel (**A**) shows the prevalence of selected high-risk clinical features across DTI groups. Panel (**B**) shows multivariable odds ratios for DTI < 15 days; the red dashed vertical line indicates OR = 1.

Patients with shorter DTI had a greater burden of adverse baseline features (Table 1; Figure 2A). Stage III–IV disease, extranodal involvement > 1 site, CNS involvement, LDH > ULN, B symptoms and IPI all showed adverse gradients with shorter DTI. Hemoglobin was also lower in the short DTI group (median 131, 145 and 139 g/L across increasing DTI groups; Kruskal–Wallis *p* = 0.014), whereas WBC, platelet count, albumin and uric acid did not differ significantly. The composite baseline disease burden score decreased across increasing DTI groups (Supplementary Figure S2).

### 3.3. Clinical Factors Associated with Short DTI

Logistic regression analyses were performed to identify clinical factors associated with DTI < 15 days (Table 2; Figure 2B). In univariable analyses, stage III–IV disease, LDH > ULN, extranodal involvement > 1 site, CNS involvement, and higher IPI score were associated with short DTI. In the multivariable model, CNS involvement remained independently associated with DTI < 15 days (OR 3.18, 95% CI 1.13–8.89, *p* = 0.028), while extranodal involvement > 1 site showed a borderline association (OR 1.79, 95% CI 0.91–3.54, *p* = 0.094).

### 3.4. Treatment Response and Early Treatment Failure

CR occurred in 46.4% of patients with DTI < 15 days, compared with 61.1% and 62.3% in the 15–29-day and ≥30-day groups, respectively. Restricted cubic spline analysis showed no clear non-linear association between DTI and the predicted probability of CR (Supplementary Figure S3). In the primary adjusted analysis treating DTI as a continuous variable, each 7-day increase in DTI was not significantly associated with CR (OR 1.04, 95% CI 0.93–1.15, *p* = 0.505). The binary 2-year outcome was ascertainable for 175 patients, of whom 74 experienced early treatment failure. Evaluable/failure counts were 49/33, 73/30 and 53/11 across the <15-day, 15–29-day and ≥30-day groups, respectively. Each 7-day increase in DTI was associated with lower odds of early treatment failure (adjusted OR 0.72, 95% CI 0.58–0.88, *p* = 0.002; Table 3). Detailed treatment and response data are provided in Supplementary Tables S3 and S4.

### 3.5. DTI and Post-Treatment Survival

**Figure 3:**
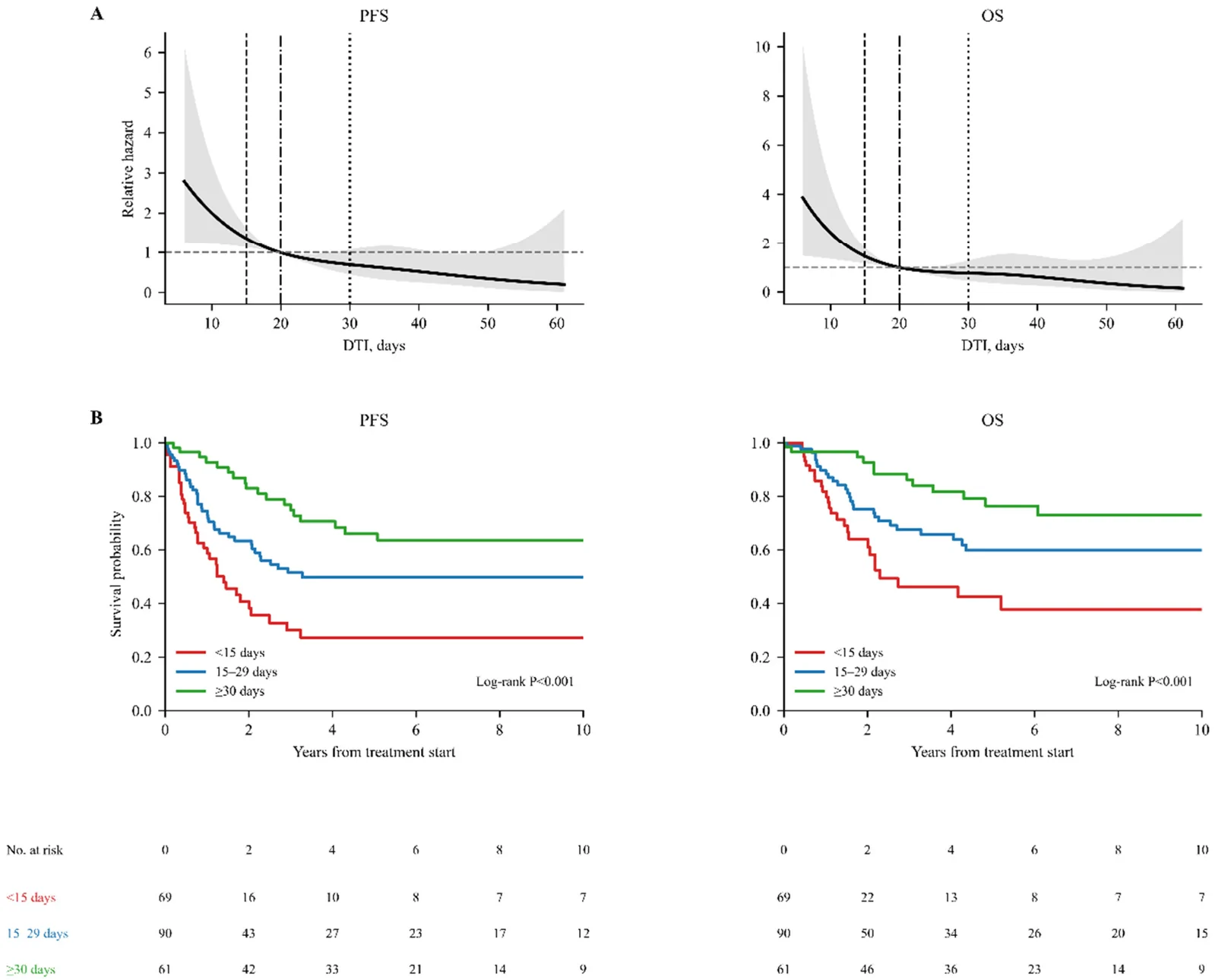
**DTI and post-treatment survival outcomes.** Panel (**A**) shows restricted cubic spline Cox models for PFS and OS from treatment start; grey shaded areas represent 95% confidence intervals. Black vertical reference lines mark DTI thresholds and the reference point: dashed line, 15 days; dash-dotted line, median DTI/reference of 20 days; dotted line, 30 days. Panel (**B**) shows Kaplan–Meier curves for PFS and OS according to DTI group, with number-at-risk tables and log-rank *p* values.

During follow-up from treatment start, 94 PFS events and 65 deaths were observed. Two-year PFS increased across DTI groups from 40.6% in the <15-day group to 63.4% and 83.0% in the 15–29-day and ≥30-day groups, respectively; corresponding two-year OS estimates were 64.1%, 75.3%, and 92.7% (Figure 3B). In primary adjusted Cox models with DTI entered as a continuous variable, each 7-day increase in DTI was associated with lower hazards of PFS events (HR 0.84, 95% CI 0.74–0.94, *p* = 0.003) and death (HR 0.84, 95% CI 0.73–0.98, *p* = 0.022; Table 3). Restricted cubic spline analyses showed the highest post-treatment PFS and OS hazards among patients with the shortest DTI, followed by a progressive decline in relative hazard with longer DTI (Figure 3A).

Categorical Cox analyses were directionally concordant with the primary continuous analysis (Table 3). Compared with the <15-day group, the adjusted HRs for PFS were 0.61 (95% CI 0.37–0.98, *p* = 0.041) in the 15–29-day group and 0.35 (95% CI 0.19–0.63, *p* < 0.001) in the ≥30-day group; for OS, the corresponding adjusted HRs were 0.61 (95% CI 0.34–1.07, *p* = 0.083) and 0.36 (95% CI 0.17–0.73, *p* = 0.005), respectively. Together with the baseline comparisons, these findings support the interpretation of shorter DTI as a marker of greater disease burden and clinical urgency rather than a protective effect of delayed treatment.

### 3.6. Sensitivity Analyses

Sensitivity analyses were directionally consistent (Supplementary Table S2). Among 162 patients with available pathology-based DTI, each 7-day increase remained associated with early treatment failure (OR 0.67, 95% CI 0.50–0.90, *p* = 0.007), PFS (HR 0.85, 95% CI 0.74–0.98, *p* = 0.021) and OS (HR 0.83, 95% CI 0.69–0.99, *p* = 0.044). The DTI group changed in 69/162 patients (42.6%) when the final pathology date replaced the biopsy date. After excluding 27 patients with documented pre-phase corticosteroid use, associations persisted for PFS (HR 0.83, 95% CI 0.74–0.94, *p* = 0.004) and OS (HR 0.77, 95% CI 0.65–0.92, *p* = 0.004). In the strictly classifiable < 25% marrow blast cohort (n = 153), associations also persisted for early failure (OR 0.66, 95% CI 0.49–0.88, *p* = 0.005), PFS (HR 0.79, 95% CI 0.67–0.93, *p* = 0.005) and OS (HR 0.81, 95% CI 0.66–0.99, *p* = 0.037). Results were likewise robust to DTI outlier exclusions, BFM-90 restriction, survival origin at biopsy and diagnosis year adjustment. Treatment era patterns are shown in Supplementary Figure S4.

## 4. Discussion

In this multicenter cohort of mass-dominant, histologically diagnosed T-LBL, shorter DTI was associated with greater clinical urgency, higher baseline disease burden, lower hemoglobin, early treatment failure and inferior post-treatment survival. The association persisted when the binary early failure analysis was restricted to patients with ascertainable 2-year status, when the final pathology date was used as an alternative DTI origin, after excluding documented pre-phase corticosteroid recipients and after restriction to patients strictly classifiable as having <25% marrow blasts. These findings support DTI as a marker of clinical urgency and disease aggressiveness rather than evidence that early treatment is harmful.

The apparently paradoxical association between shorter DTI and inferior outcomes has been increasingly recognized in aggressive lymphomas [12,13]. In DLBCL, patients treated soon after diagnosis often have inferior survival despite earlier therapy [12,15,17]. Maurer et al. showed that DTI was an important clinical factor in newly diagnosed DLBCL and highlighted its relevance to time-dependent selection bias in clinical trials [12]. Population-based data similarly linked shorter DTI to inferior OS in DLBCL, supporting the view that patients requiring urgent treatment represent a clinically higher risk population [17]. Pretreatment ctDNA studies further clarified this association by showing that short DTI correlates with advanced-stage disease, higher IPI, greater metabolic tumor volume and higher ctDNA levels, suggesting that DTI captures baseline tumor burden and aggressive biology [21]. Our findings extend this concept to T-LBL, a rare aggressive lymphoma in which urgent treatment is common, but the prognostic meaning of DTI has not been systematically studied.

The relationship between DTI and outcome should therefore be interpreted within the framework of confounding by indication. In routine practice, patients with bulky or rapidly progressive disease, CNS involvement, organ compromise, anemia or other high-risk features are more likely to receive immediate treatment, while these same features increase the risk of treatment failure and death [12,21]. In our cohort, CNS involvement remained independently associated with DTI < 15 days, and the lower hemoglobin observed in the short-DTI group provides additional evidence of baseline disease burden. DTI should therefore not be used to justify delay; short DTI identifies patients who require urgent treatment yet remain at high risk.

Our results also align with broader evidence that the meaning of treatment intervals differs across lymphoma subtypes [19]. A recent systematic review reported substantial heterogeneity in definitions, distributions and prognostic associations of diagnostic and treatment intervals in lymphoma [19]. Several studies in aggressive lymphomas described non-linear or paradoxical associations, with worse outcomes among patients with very short treatment intervals, likely reflecting acute presentation and aggressive biology [12,13,21,23,24,25]. The review also emphasized the limitations of arbitrary dichotomization and recommended continuous analyses with evaluation of non-linear relationships when possible [19]. Accordingly, we analyzed DTI primarily as a continuous variable per 7-day increase, used restricted cubic splines to explore non-linearity, and retained categorical DTI groups only for descriptive comparison, visualization and clinical interpretability.

Findings from other lymphoma subtypes further illustrate the context-dependent nature of DTI [19]. In mantle cell lymphoma, DTI has been associated with clinical characteristics and outcomes, supporting its relevance beyond DLBCL [16]. Conversely, recent marginal zone lymphoma data suggest that short DTI may not necessarily translate into inferior survival despite associations with selected adverse features [18]. These differences are biologically plausible given variations in disease tempo and reasons for treatment initiation. T-LBL is a highly aggressive precursor T-cell neoplasm that often presents with advanced disease, mediastinal involvement, extranodal dissemination, marrow involvement or CNS disease [1,2]. In this setting, a short interval from diagnosis to therapy is more likely to represent clinical urgency than efficient care alone.

The dissociation between DTI and CR versus DTI and PFS/OS/early failure is clinically informative. CR mainly reflects initial chemosensitivity, whereas PFS, OS and early failure also capture relapse driven by underlying disease biology. Short DTI patients may achieve an initial response yet remain at increased relapse risk. DTI is therefore a readily available urgency marker that may inform early monitoring and cohort characterization, and it should be reported when comparing real-world cohorts or trials whose screening procedures may exclude patients requiring immediate treatment.

These findings have several clinical implications. First, DTI is a readily available real-world marker of disease urgency in T-LBL. It requires no additional testing and captures clinical decision-making information not fully represented by conventional baseline variables. Second, short DTI may identify patients who warrant heightened early monitoring, supportive care and careful response assessment, because rapid treatment initiation does not eliminate the adverse risk associated with aggressive presentation. Third, DTI should be considered when comparing retrospective cohorts or prospective trials. Lengthy trial screening procedures may preferentially exclude patients requiring immediate treatment, thereby enriching trial populations for more stable patients and potentially limiting generalizability [12]. Reporting DTI may help characterize such selection effects.

This study has limitations. First, the retrospective design introduced potential selection bias and residual confounding, and treatment timing was determined by real-world clinical practice rather than standardized protocols, precluding causal inference regarding the association between DTI and outcomes. The exclusion of patients who received pretreatment before referral may also have preferentially omitted clinically unstable cases. Second, although the diagnostic biopsy date was used as a uniform operational baseline, interinstitutional differences in diagnostic workflows, referral processes, and pathological confirmation could not be fully captured. In addition, the 25% bone marrow blast threshold separating T-LBL from T-ALL is operational rather than biologically defined; therefore, caution is warranted when extrapolating our findings to strictly marrow-defined T-LBL, although results remained consistent among patients with marrow blasts < 25%. Third, pre-phase therapy, emergency cytoreduction, and some baseline clinical data were not uniformly documented, and treatment intensity, dose modification, adherence, and consolidation strategies were not fully incorporated into the primary models. Hematopoietic stem cell transplantation was not treated as a baseline covariate because of its time-dependent nature and potential for immortal time bias. Fourth, the absence of uniformly available molecular biomarkers, measurable residual disease, metabolic tumor burden, and ctDNA data limited the biological validation of DTI as a marker of disease aggressiveness. Finally, the rarity of T-LBL and uneven case distribution across centers restricted extensive subgroup and center-specific analyses. Nevertheless, multiple sensitivity analyses yielded consistent results, supporting the stability of the main findings.

## 5. Conclusions

In this multicenter cohort of mass-dominant, histologically diagnosed T-LBL, shorter DTI was associated with higher baseline disease burden, greater clinical urgency, increased early treatment failure, and inferior post-treatment survival. When analyzed as a continuous variable, each 7-day increase in DTI was associated with lower odds of early treatment failure and lower hazards of PFS events and death. These findings suggest that DTI in T-LBL should be interpreted primarily as a pragmatic real-world marker of clinical urgency and disease burden rather than as a simple measure of treatment delay. Rather than serving as a standalone decision-making tool, DTI may be useful for cohort characterization, interpretation of real-world outcomes and assessment of clinical trial generalizability.

## Figures and Tables

**Table 1:** Baseline characteristics according to the DTI group.

Variable	<15 Days (n = 69)	15–29 Days (n = 90)	≥30 Days (n = 61)	*p* Value	*p* for Trend
Age, median (IQR)	24 (18–35)	26 (18–38)	29 (20–40)	0.305	
Male sex	52 (75.4%)	62 (68.9%)	43 (70.5%)	0.660	
Stage III–IV	62 (89.9%)	74 (82.2%)	44 (72.1%)	0.032	0.010
LDH > ULN	33 (47.8%)	32 (35.6%)	18 (29.5%)	0.085	0.031
WBC, ×10^9^/L	5.48 (3.68–7.00)	6.60 (4.59–9.23)	6.32 (4.60–7.33)	0.063	
Hemoglobin, g/L	131 (99–149)	145 (128–158)	139 (125–152)	0.014	
Platelet count, ×10^9^/L	211 (115–252)	232 (169–272)	230 (187–286)	0.097	
Albumin, g/L	43.8 (38.0–46.2)	44.1 (41.5–47.7)	44.1 (42.2–48.8)	0.280	
Uric acid, μmol/L	365 (292–455)	357 (296–440)	348 (272–425)	0.547	
Ki-67 ≥ 75%	44 (63.8%)	60 (66.7%)	31 (50.8%)	0.129	
B symptoms	23 (33.3%)	24 (26.7%)	10 (16.4%)	0.087	0.030
ECOG > 1	16 (23.2%)	10 (11.1%)	12 (19.7%)	0.115	
Extranodal involvement > 1 site	48 (69.6%)	46 (51.1%)	26 (42.6%)	0.006	0.002
Bone marrow involvement	37 (53.6%)	48 (53.3%)	25 (41.0%)	0.253	
CNS involvement	12 (17.4%)	6 (6.7%)	1 (1.6%)	0.004	0.003
Mediastinal involvement	44 (63.8%)	64 (71.1%)	39 (63.9%)	0.531	
Bulky disease > 7.5 cm	23 (33.3%)	30 (33.3%)	17 (27.9%)	0.738	
IPI score, median (IQR)	3 (2–3)	2 (1–3)	2 (1–3)	0.001	<0.001

Abbreviations: CNS, central nervous system; DTI, diagnosis-to-treatment interval; ECOG, Eastern Cooperative Oncology Group; IPI, International Prognostic Index; IQR, interquartile range; LDH, lactate dehydrogenase; ULN, upper limit of normal; WBC, white blood cell count. Continuous laboratory measures are presented as median (IQR) and were compared using Kruskal–Wallis tests. WBC, hemoglobin, platelet count, albumin and uric acid were available in 176 patients. *p* for trend is shown only for variables with a prespecified ordinal gradient.

**Table 2:** Clinical factors associated with DTI < 15 days.

Variable	Univariable OR (95% CI)	Univariable *p*	Multivariable OR (95% CI)	Multivariable *p*
Age per 10-year increase	0.89 (0.71–1.11)	0.301		
Male sex	1.34 (0.70–2.56)	0.376		
Stage III–IV	2.48 (1.04–5.92)	0.041	1.71 (0.65–4.51)	0.277
LDH > ULN	1.85 (1.04–3.31)	0.038	1.27 (0.68–2.40)	0.455
Ki-67 ≥ 75%	1.16 (0.64–2.09)	0.621		
B symptoms	1.72 (0.92–3.23)	0.091	1.55 (0.80–3.02)	0.195
ECOG > 1	1.77 (0.86–3.63)	0.120		
Extranodal involvement > 1 site	2.51 (1.37–4.59)	0.003	1.79 (0.91–3.54)	0.094
Bone marrow involvement	1.24 (0.70–2.19)	0.468		
CNS involvement	4.33 (1.62–11.55)	0.003	3.18 (1.13–8.89)	0.028
Mediastinal involvement	0.82 (0.45–1.49)	0.516		
Bulky disease > 7.5 cm	1.11 (0.60–2.03)	0.744		
IPI score	1.54 (1.19–1.99)	<0.001		

Abbreviations: CI, confidence interval; CNS, central nervous system; DTI, diagnosis-to-treatment interval; ECOG, Eastern Cooperative Oncology Group; LDH, lactate dehydrogenase; OR, odds ratio; ULN, upper limit of normal. Odds ratios are for DTI < 15 days. The multivariable model included stage III–IV disease, LDH > ULN, B symptoms, extranodal involvement > 1 site, and CNS involvement.

**Table 3:** Association of DTI with treatment response, early treatment failure and survival outcomes.

Outcome	Analysis	Comparison	Measure	Effect Estimate (95% CI)	*p* Value	Model
Complete response	Primary continuous analysis	DTI per 7-day increase	OR	1.04 (0.93–1.15)	0.505	Adjusted logistic
Early treatment failure within 2 years	Primary continuous analysis	DTI per 7-day increase	OR	0.72 (0.58–0.88)	0.002	Adjusted logistic
Progression-free survival	Primary continuous analysis	DTI per 7-day increase	HR	0.84 (0.74–0.94)	0.003	Adjusted Cox
Overall survival	Primary continuous analysis	DTI per 7-day increase	HR	0.84 (0.73–0.98)	0.022	Adjusted Cox
Complete response	Categorical analysis	15–29 days vs. <15 days	OR	1.69 (0.86–3.33)	0.127	Adjusted logistic
Complete response	Categorical analysis	≥30 days vs. <15 days	OR	1.98 (0.92–4.26)	0.080	Adjusted logistic
Early treatment failure within 2 years	Categorical analysis	15–29 days vs. <15 days	OR	0.43 (0.18–1.04)	0.062	Adjusted logistic
Early treatment failure within 2 years	Categorical analysis	≥30 days vs. <15 days	OR	0.14 (0.05–0.42)	<0.001	Adjusted logistic
Progression-free survival	Categorical analysis	15–29 days vs. <15 days	HR	0.61 (0.37–0.98)	0.041	Adjusted Cox
Progression-free survival	Categorical analysis	≥30 days vs. <15 days	HR	0.35 (0.19–0.63)	<0.001	Adjusted Cox
Overall survival	Categorical analysis	15–29 days vs. <15 days	HR	0.61 (0.34–1.07)	0.083	Adjusted Cox
Overall survival	Categorical analysis	≥30 days vs. <15 days	HR	0.36 (0.17–0.73)	0.005	Adjusted Cox

Abbreviations: CI, confidence interval; CNS, central nervous system; DTI, diagnosis-to-treatment interval; ECOG, Eastern Cooperative Oncology Group; HR, hazard ratio; LDH, lactate dehydrogenase; OR, odds ratio; OS, overall survival; PFS, progression-free survival. Primary analyses report effects per 7-day DTI increase. Categorical analyses use DTI <15 days as reference. Adjusted models included age, stage, ECOG, LDH, extranodal involvement >1 site, bone marrow involvement and CNS involvement. The binary early treatment failure analysis included 175 patients with ascertainable 2-year status and 74 events.

## Data Availability

Data supporting the findings of this study are available from the corresponding author upon reasonable request.

## Supplementary Materials

The following supporting information can be downloaded at: https://www.mdpi.com/article/10.3390/jcm15166408/s1, Supplementary Figure S1: Patient flow diagram showing screening, reason-specific and center-level exclusions, and the final analytic cohort; Supplementary Figure S2: Disease burden score by DTI group. Boxes show the IQR, center lines the median, whiskers values within 1.5 × IQR and open circles outliers. Kruskal–Wallis *p* < 0.001; Supplementary Figure S3: Association between DTI and complete response. Restricted cubic spline logistic regression shows predicted CR probability; shading indicates 95% CI and dashed vertical lines indicate 15 and 30 days; Supplementary Figure S4: DTI and outcomes across treatment eras. Panels show DTI by year, DTI by era, and PFS and OS by era; Supplementary Table S1. Data availability and missingness. Supplementary Table S2. Sensitivity analyses for DTI and outcomes. Supplementary Table S3. Treatment patterns and response according to DTI group. Supplementary Table S4. Full logistic models for treatment response and early treatment failure. Supplementary Table S5. Full Cox models for PFS and OS.
