# Supplementary material for: Diagnosis-to-Treatment Interval Reflects Clinical Urgency and Disease Burden in T-Lymphoblastic Lymphoma: A Multicenter Cohort Study
Source: J Clin Med. 2026 Aug 19;15(16):6408. doi: 10.3390/jcm15166408 (PMC13513362; doi:10.3390/jcm15166408)
Supplement: Supplementary file 1 [file jcm-15-06408-s001.zip › Supplementary_Table_S5.pdf]

Supplementary Table S5. Full Cox models for PFS and OS.

Endpoint	Model	Comparison	Events/N	HR (95% CI)	P value
PFS from treatment start	Model 1: DTI only	15–29 days vs <15 days	94/220	0.53 (0.33-0.83)	0.005
PFS from treatment start	Model 1: DTI only	≥30 days vs <15 days	94/220	0.28 (0.16-0.49)	<0.001
PFS from treatment start	Model 2: + age/stage/ECOG/LDH	15–29 days vs <15 days	94/220	0.62 (0.39-0.98)	0.043
PFS from treatment start	Model 2: + age/stage/ECOG/LDH	≥30 days vs <15 days	94/220	0.32 (0.18-0.56)	<0.001
PFS from treatment start	Model 3: + disease burden	15–29 days vs <15 days	94/220	0.61 (0.37-0.98)	0.041
PFS from treatment start	Model 3: + disease burden	≥30 days vs <15 days	94/220	0.35 (0.19-0.63)	<0.001
OS from treatment start	Model 1: DTI only	15–29 days vs <15 days	65/220	0.52 (0.31-0.90)	0.020
OS from treatment start	Model 1: DTI only	≥30 days vs <15 days	65/220	0.27 (0.14-0.54)	<0.001
OS from treatment start	Model 2: + age/stage/ECOG/LDH	15–29 days vs <15 days	65/220	0.60 (0.35-1.04)	0.071
OS from treatment start	Model 2: + age/stage/ECOG/LDH	≥30 days vs <15 days	65/220	0.32 (0.16-0.65)	0.001
OS from treatment start	Model 3: + disease burden	15–29 days vs <15 days	65/220	0.61 (0.34-1.07)	0.083
OS from treatment start	Model 3: + disease burden	≥30 days vs <15 days	65/220	0.36 (0.17-0.73)	0.005

Hazard ratios compare each DTI group with DTI <15 days. Survival time was calculated from treatment start.
