# Supplementary material for: Diagnosis-to-Treatment Interval Reflects Clinical Urgency and Disease Burden in T-Lymphoblastic Lymphoma: A Multicenter Cohort Study
Source: J Clin Med. 2026 Aug 19;15(16):6408. doi: 10.3390/jcm15166408 (PMC13513362; doi:10.3390/jcm15166408)
Supplement: Supplementary file 1 [file jcm-15-06408-s001.zip › Supplementary_Figure_Captions.pdf]

Supplementary Figure Captions

Supplementary Figure S1. Patient flow diagram. Flow diagram showing cohort assembly from initial screening (n = 236, four centers) to the final analytic cohort (n = 220). Exclusion reasons (n = 16) and center distribution are detailed.

Supplementary Figure S2. Disease burden score by diagnosis-to-treatment interval (DTI) group. Disease burden score was defined as the sum of six baseline adverse features, assigning one point each for stage III–IV disease, lactate dehydrogenase (LDH) above the upper limit of normal (ULN), Eastern Cooperative Oncology Group (ECOG) performance status > 1, extranodal involvement > 1 site, bone marrow involvement, and central nervous system (CNS) involvement. The box represents the interquartile range (IQR; 25th–75th percentile); the horizontal line represents the median; whiskers extend to 1.5 × IQR; individual data points are shown as jittered dots. Kruskal–Wallis test $p < 0.001$ across DTI groups.

Supplementary Figure S3. Association between diagnosis-to-treatment interval (DTI) and complete response. Restricted cubic spline logistic regression shows the predicted probability of complete response according to DTI, with the display truncated at the 95th percentile of DTI. Grey shaded areas represent 95% confidence intervals; black dashed vertical reference lines indicate 15 and 30 days.

Supplementary Figure S4. Diagnosis-to-treatment interval (DTI) and outcomes across treatment eras. Panel A shows the distribution of DTI by year of diagnosis with locally estimated scatterplot smoothing (LOESS); the grey band represents the 95% confidence interval (CI), and the dashed line indicates the cohort median DTI of 20 days. Panel B shows DTI according to treatment era (2003–2010, 2011–2017, and 2018–2025). Panels C and D show Kaplan–Meier curves for progression-free survival (PFS) and overall survival (OS), respectively, according to treatment era, with number-at-risk tables.
