# Supplementary material for: Diagnosis-to-Treatment Interval Reflects Clinical Urgency and Disease Burden in T-Lymphoblastic Lymphoma: A Multicenter Cohort Study
Source: J Clin Med. 2026 Aug 19;15(16):6408. doi: 10.3390/jcm15166408 (PMC13513362; doi:10.3390/jcm15166408)
Supplement: Supplementary file 1 [file jcm-15-06408-s001.zip › Supplementary_Figure_S4.pdf]

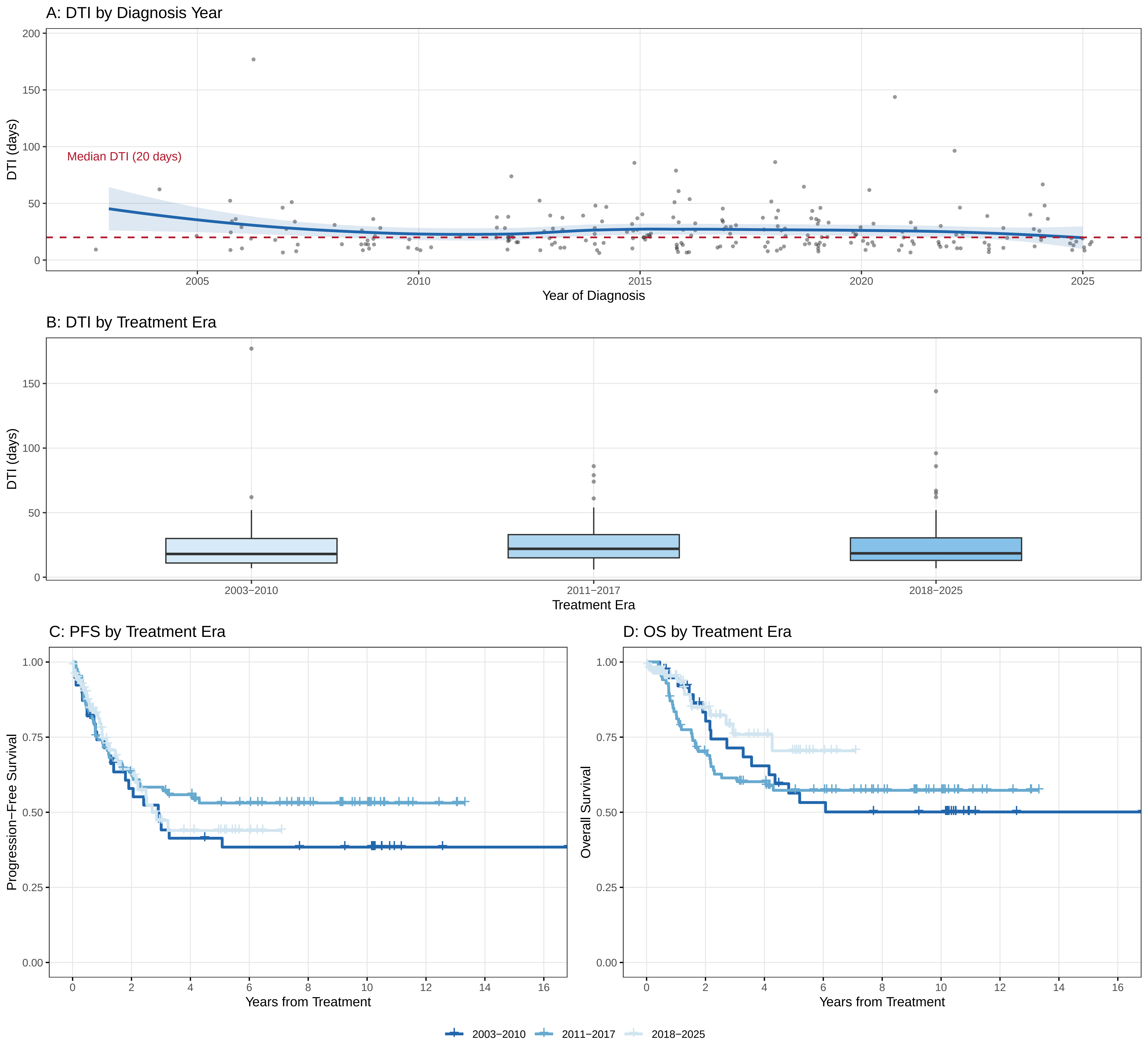

A: DTI by Diagnosis Year
DTI (days)
Median DTI (20 days)
Year of Diagnosis
2005
2010
2015
2020
2025
0
50
100
150
200
B: DTI by Treatment Era
DTI (days)
Treatment Era
2003−2010
2011−2017
2018−2025
C: PFS by Treatment Era
Progression−Free Survival
Years from Treatment
0.00
0.25
0.50
0.75
1.00
0
2
4
6
8
10
12
14
16
D: OS by Treatment Era
Overall Survival
Years from Treatment
2003−2010
2011−2017
2018−2025
