# Supplementary material for: Diagnosis-to-Treatment Interval Reflects Clinical Urgency and Disease Burden in T-Lymphoblastic Lymphoma: A Multicenter Cohort Study
Source: J Clin Med. 2026 Aug 19;15(16):6408. doi: 10.3390/jcm15166408 (PMC13513362; doi:10.3390/jcm15166408)
Supplement: Supplementary file 1 [file jcm-15-06408-s001.zip › Supplementary_Table_S1.pdf]

Supplementary Table S1. Data availability and missingness.

Variable	Available, n	Missing/unknown, n	Available, %
Core baseline and outcome variables used in primary models	220	0	100.0
Bone marrow blast percentage among 110 with marrow involvement	97	13	88.2
WBC	176	44	80.0
Hemoglobin	176	44	80.0
Platelet count	176	44	80.0
Albumin	176	44	80.0
Uric acid	176	44	80.0
Pathology-based DTI	162	58	73.6
Prephase corticosteroid status	59	161	26.8
Prephase start date among 27 documented recipients	23	4	85.2

Core model variables were complete and primary models used all eligible observations. Supplementary variables were analyzed using available cases without imputation; their availability is reported above.
