# Supplementary material for: Diagnosis-to-Treatment Interval Reflects Clinical Urgency and Disease Burden in T-Lymphoblastic Lymphoma: A Multicenter Cohort Study
Source: J Clin Med. 2026 Aug 19;15(16):6408. doi: 10.3390/jcm15166408 (PMC13513362; doi:10.3390/jcm15166408)
Supplement: Supplementary file 1 [file jcm-15-06408-s001.zip › Supplementary_Table_S2.pdf]

Supplementary Table S2. Sensitivity analyses for DTI and outcomes.

Sensitivity analysis	Endpoint	Comparison	N/events	Adjusted effect (95% CI)	P value
Primary analysis	PFS	DTI per 7-day increase	220/94	HR 0.84 (0.74–0.94)	0.003
Primary analysis	OS	DTI per 7-day increase	220/65	HR 0.84 (0.73–0.98)	0.022
Exclude DTI >60 days	PFS	DTI per 7-day increase	208/90	HR 0.77 (0.66–0.90)	0.001
Exclude DTI >60 days	OS	DTI per 7-day increase	208/62	HR 0.76 (0.62–0.92)	0.005
Exclude DTI >90 days	PFS	DTI per 7-day increase	217/94	HR 0.84 (0.75–0.95)	0.005
Exclude DTI >90 days	OS	DTI per 7-day increase	217/65	HR 0.85 (0.73–0.98)	0.030
BFM-90-treated only	PFS	DTI per 7-day increase	201/89	HR 0.83 (0.74–0.94)	0.004
BFM-90-treated only	OS	DTI per 7-day increase	201/64	HR 0.84 (0.72–0.98)	0.023
Survival time from biopsy	PFS	DTI per 7-day increase	220/94	HR 0.83 (0.74–0.93)	0.002
Survival time from biopsy	OS	DTI per 7-day increase	220/65	HR 0.84 (0.72–0.97)	0.017
Additional diagnosis-year adjustment	PFS	DTI per 7-day increase	220/94	HR 0.83 (0.74–0.94)	0.002
Additional diagnosis-year adjustment	OS	DTI per 7-day increase	220/65	HR 0.84 (0.73–0.97)	0.020
Pathology-based DTI	Early failure	DTI per 7-day increase	126/52	OR 0.67 (0.50–0.90)	0.007
Pathology-based DTI	PFS	DTI per 7-day increase	162/71	HR 0.85 (0.74–0.98)	0.021
Pathology-based DTI	OS	DTI per 7-day increase	162/47	HR 0.83 (0.69–0.99)	0.044
Exclude documented prephase corticosteroids	PFS	DTI per 7-day increase	193/84	HR 0.83 (0.74–0.94)	0.004
Exclude documented prephase corticosteroids	OS	DTI per 7-day increase	193/58	HR 0.77 (0.65–0.92)	0.004
Strictly classifiable marrow blasts <25%	Early failure	DTI per 7-day increase	123/42	OR 0.66 (0.49–0.88)	0.005
Strictly classifiable marrow blasts <25%	PFS	DTI per 7-day increase	153/58	HR 0.79 (0.67–0.93)	0.005
Strictly classifiable marrow blasts <25%	OS	DTI per 7-day increase	153/41	HR 0.81 (0.66–0.99)	0.037

Effects are adjusted for age, stage, ECOG, LDH, extranodal involvement >1 site, bone marrow involvement and CNS involvement. Pathology-based DTI analyses used 162 patients in whom this DTI was available. Binary early-failure analyses were restricted to patients with ascertainable 2-year status.
