# Supplementary material for: Diagnosis-to-Treatment Interval Reflects Clinical Urgency and Disease Burden in T-Lymphoblastic Lymphoma: A Multicenter Cohort Study
Source: J Clin Med. 2026 Aug 19;15(16):6408. doi: 10.3390/jcm15166408 (PMC13513362; doi:10.3390/jcm15166408)
Supplement: Supplementary file 1 [file jcm-15-06408-s001.zip › Supplementary_Table_S3.pdf]

Supplementary Table S3. Treatment patterns and response according to DTI group.

Variable	<15 days (n=69)	15–29 days (n=90)	≥30 days (n=61)	P value	P for trend
BFM-90 first-line	60 (87.0%)	85 (94.4%)	56 (91.8%)	0.247	0.304
Complete response	32 (46.4%)	55 (61.1%)	38 (62.3%)	0.106	0.063
Partial response	27 (39.1%)	23 (25.6%)	20 (32.8%)	0.187	0.402
Stable disease	6 (8.7%)	7 (7.8%)	3 (4.9%)	0.690	0.416
Progressive disease	4 (5.8%)	5 (5.6%)	0 (0.0%)	0.165	0.116
Overall response	59 (85.5%)	78 (86.7%)	58 (95.1%)	0.171	0.096
Any HSCT	12 (17.4%)	33 (36.7%)	19 (31.1%)	0.027	0.073
Intrathecal chemotherapy	55 (79.7%)	67 (74.4%)	46 (75.4%)	0.725	0.550
Radiotherapy	5 (7.2%)	3 (3.3%)	4 (6.6%)	0.507	0.828

This table includes treatment-related variables and detailed response categories that were not included in the baseline characteristics table.
