# Supplementary material for: Diagnosis-to-Treatment Interval Reflects Clinical Urgency and Disease Burden in T-Lymphoblastic Lymphoma: A Multicenter Cohort Study
Source: J Clin Med. 2026 Aug 19;15(16):6408. doi: 10.3390/jcm15166408 (PMC13513362; doi:10.3390/jcm15166408)
Supplement: Supplementary file 1 [file jcm-15-06408-s001.zip › Supplementary_Table_S4.pdf]

Supplementary Table S4. Full logistic models for treatment response and early treatment failure.

Outcome	Model	Comparison	OR (95% CI)	P value
Complete response	Univariable	15–29 days vs <15 days	1.82 (0.96-3.43)	0.065
Complete response	Univariable	≥30 days vs <15 days	1.91 (0.95-3.85)	0.071
Complete response	Adjusted	15–29 days vs <15 days	1.69 (0.86-3.33)	0.127
Complete response	Adjusted	≥30 days vs <15 days	1.98 (0.92-4.26)	0.080
Overall response	Univariable	15–29 days vs <15 days	1.10 (0.45-2.72)	0.834
Overall response	Univariable	≥30 days vs <15 days	3.28 (0.86-12.52)	0.083
Overall response	Adjusted	15–29 days vs <15 days	0.98 (0.36-2.63)	0.962
Overall response	Adjusted	≥30 days vs <15 days	3.87 (0.91-16.37)	0.066
Early treatment failure within 2 years	Univariable	15–29 days vs <15 days	0.34 (0.16–0.72)	0.005
Early treatment failure within 2 years	Univariable	≥30 days vs <15 days	0.13 (0.05–0.31)	<0.001
Early treatment failure within 2 years	Adjusted	15–29 days vs <15 days	0.43 (0.18–1.04)	0.062
Early treatment failure within 2 years	Adjusted	≥30 days vs <15 days	0.14 (0.05–0.42)	<0.001

Odds ratios compare each DTI group with DTI <15 days. Early-treatment-failure models included 175 patients with ascertainable 2-year status and 74 events.
